# Effects of Rotations With Legume on Soil Functional Microbial Communities Involved in Phosphorus Transformation

**DOI:** 10.3389/fmicb.2021.661100

**Published:** 2021-09-30

**Authors:** Hui Yu, Fenghua Wang, Minmin Shao, Ling Huang, Yangyang Xie, Yuxin Xu, Lingrang Kong

**Affiliations:** ^1^National Engineering Laboratory for Efficient Utilization of Soil and Fertilizer Resources, College of Resources and the Environment, Shandong Agricultural University, Taian, China; ^2^State Key Laboratory of Crop Biology, College of Agronomy, Shandong Agricultural University, Taian, China; ^3^Jining Academy of Agricultural Sciences, Jining, China

**Keywords:** soybean-wheat rotation, maize-wheat rotation, phosphorus cycling genes, metagenome, *gcd* gene

## Abstract

Including legumes in the cereal cropping could improve the crop yield and the uptake of nitrogen (N) and phosphorus (P) of subsequent cereals. The effects of legume-cereal crop rotations on the soil microbial community have been studied in recent years, the impact on soil functional genes especially involved in P cycling is raising great concerns. The metagenomic approach was used to investigate the impacts of crop rotation managements of soybean-wheat (SW) and maize-wheat (MW) lasting 2 and 7years on soil microbial communities and genes involved in P transformation in a field experiment. Results indicated that SW rotation increased the relative abundances of *Firmicutes* and *Bacteroidetes*, reduced *Actinobacteria*, *Verrucomicrobia*, and *Chloroflexi* compared to MW rotation. *gcd*, *phoR*, *phoD*, and *ppx* predominated in genes involved in P transformation in both rotations. Genes of *gcd*, *ppa*, and *ugpABCE* showed higher abundances in SW rotation than in MW rotation, whereas *gadAC* and *pstS* showed less abundances. *Proteobacteria*, *Acidobacteria*, and *Gemmatimonadetes* played predominant roles in microbial P cycling. Our study provides a novel insight into crop P, which requires strategy and help to understand the mechanism of improving crop nutrient uptake and productivity in different rotations.

## Introduction

Crop rotation could improve soil properties, depress pathogens, and shift the soil microbial community composition and diversity both in bulk and rhizosphere soils (Jiang et al., 2016; Liu et al., 2017; Soman et al., 2017). Introducing legumes in crop rotation improved soil physical, chemical, and biological properties (Aschi et al., 2017), and enhanced C and N accumulation rates through increased root diversity. Furthermore, legume-cereal rotation had more profound influence on soil microbial community than cereal-cereal rotation did (Wang et al., 2017). For example, inclusion of pulses in the maize-wheat rotation improved soil organic carbon (SOC), plant available nitrogen (AN) and available phosphorus (AP) and enhanced the nutrient uptake in a maize crop (Venkatesh et al., 2017).

Soil microorganisms play an important role in the cycling of N and P and improving their availability to crops. There are more studies on the effects of crop rotation, especially legumes-cereal rotation, on soil microbial communities. The preceding chickpea enhanced the colonization of arbuscular mycorrhizal fungi (AMF) in wheat root than that of canola rotation and increased the wheat yield (Bakhshandeh et al., 2017). The result of a phospholipid fatty acid (PLFA) profile suggested that short-term (2years) crop rotation had more effects on soil fungal communities than on bacteria and reduced the fungal biomass, especially AMF (Zhang et al., 2014). Crop management practices with 13years of crop succession [soybean (summer)/wheat (winter)] or rotation [soybean/maize (summer)/wheat/lupine/oat (winter)] indicated that crop rotation increased the abundance of *Actinomycetales* (*Actinobacteria*), *Solibacterales*, and *Acidobacteriales*, and decreased *Sphingomonadales* and *Chloroflexales* (Souza et al., 2013). In wheat-soybean rotation, soybean planting increased the relative abundance of *Firmicutes* in bacteria and *Glomeromycota* in fungi (Guo et al., 2020). Also, the function of soil microbial community, especially those involved in C, N, and P cycling, were explored in some studies (Paungfoo-Lonhienne et al., 2017; Soman et al., 2017; Linton et al., 2020; Shen et al., 2021). Metagenomic sequences indicated that different crop managements between soybean (summer)/wheat (winter) and soybean/maize (summer)/wheat/lupine/oat (winter) in a 13-year-old field experiment in southern Brazil affected microbial function, the latter had more genes of amino acids and derivatives and carbohydrate subsystems metabolism (Souza et al., 2015).

P is an essential element for all forms of life and is a crucial resource for all growth. It cannot be replaced or manufactured to ensure food security. Now humanity faces two unprecedented problems. One is the scarcity of P as a nonrenewable resource (Neset and Cordell, 2012; Alewell et al., 2020). Global P resources may be exhausted in the next 40–400years (Van Kauwenbergh, 2010; Johnston et al., 2014). Therefore, P scarcity is emerging as one of the twenty-first century’s greatest global sustainability challenges. The other is an oversupply of P fertilizers resulting in eutrophication (MacDonald et al., 2011; Li et al., 2014). P is prone to accumulate in the soil, and the recycling efficiency of P for crop uptake and utility is low. Therefore, P is described as “life’s bottleneck” by Isaac Asimov (Cordell and White, 2014). Some legumes can mobilize less-labile forms of P by exudating organic acid with small molecules such as malic acid, oxalic acid, and citric acid (Richardson et al., 2009; Mat Hassan et al., 2012) and phosphatase into available P. They can also access to more of the labile P through a developed root architecture (Vandamme et al., 2013) to enhance the P uptake of the subsequent cereal. The preceding legume also raised cereal shoot P concentration and P uptake through higher arbuscular mycorrhizal (AM) infection rates in cereal (Alvey et al., 2001). Thus, introduction of legumes in cereal rotation is helpful for improving P utilization efficiency in subsequent cereal. Consequently, it is essential to use various rotation management measures to shape soil microbial community and function and then enhance P transformation and uptake by crops for sustainable agricultural development (Merloti et al., 2019; Walkup et al., 2020).

To date, microbial functions in agricultural soil are focusing on C and N metabolism and cycling (Munroe et al., 2016; Smith et al., 2016). Little is understood about how functional genes and microorganisms involved in P metabolism and transformation are affected by crop rotation management. The research can help to better understand how P is transformed and the P requirement strategy of crops. In the present study, metagenomic sequencing was used to (1) investigate the effects of legume-cereal rotation on soil microbial community structure; and (2) investigate the effects of legume-cereal rotation on soil microbial functional genes involved in P cycling.

## Materials and Methods

### The Experiment Design and Soil Sampling

Soil samples were taken from the wheat field in Jining Academy of Agricultural Sciences (35°27′41″ N, 116°35′34″E), Jining City, Shandong Province, China. The two rotations were maize-wheat rotation (MW) and soybean-wheat rotation (SW) and both lasted 7 and 2years, respectively. Therefore, the samples were designated as MW7, MW2 and SW7 and SW2. The experiment was conducted in a completely randomized block design with three replications for each treatment with a plot area of 8m×3m. In the maize season in MW rotation, the N fertilizer was provided as urea at 207kgNha^−1^, P and potassium fertilizer was potassium dihydrogen phosphate at 58.5kg P ha^−1^ and 38.3kgKha^−1^ before sowing maize, and 42kg of N ha^−1^ (urea) was applied as top dressing at the trumpet stage of maize. In the wheat season, the plots were treated with N (151.2kgha^−1^, applied as urea), P (52.4kgha^−1^, applied as diammonium hydrogen phosphate), and K (117.8kgha^−1^ applied as potassium chloride) as a base, and 120kgha^−1^ of N as urea applied as top dressing at the shooting stage of winter wheat. The field in SW rotation was fertilized with N (22.5kgha^−1^), P (10kgha^−1^), and K (18.7kgha^−1^) in soybean season, and with N (82.5kgha^−1^), P (36.7kgha^−1^), and K (68.6kgha^−1^) as a base and 155.3kg a^−1^of N (urea) as top dressing at the shooting stage of winter wheat. The mean annual precipitation and temperature are 600–800mm and 13–14°C, respectively. According to the World Reference Base for Soil Resources (WRB) the soil is classified as argosols. The soil physical and chemical properties are shown in Table 1.

**Table 1:** Soil properties related to crop rotations.

Rotation type	MW7	MW2	SW7	SW2
SOC (g·kg^−1^)	16.24 ± 0.064a	13.438 ± 0.396c	16.825 ± 0.776a	14.718 ± 0.445b
TN (g·kg^−1^)	1.048 ± 0.042a	0.866 ± 0.048b	1.048 ± 0.038a	1.103 ± 0.059a
TP (g·kg^−1^)	0.632 ± 0.038b	0.703 ± 0.028b	0.670 ± 0.043b	0.980 ± 0.031a
TK (g·kg^−1^)	12.214 ± 0.433a	11.431 ± 0.135b	12.172 ± 0.092a	12.085 ± 0.115a
pH	6.6 ± 0.1a	6.5 ± 0.1a	7 ± 0.2a	6.7 ± 0.1a
AN (mg·kg^−1^)	113.38 ± 3.61a	33.634 ± 0.764d	55.292 ± 5.937c	83.829 ± 4.479b
AP (mg·kg^−1^)	96.95 ± 1.04b	104.571 ± 1.338a	90.581 ± 0.465c	73.356 ± 1.419d
AK (mg·kg^−1^)	268.92 ± 20.00b	126.360 ± 1.645d	158.660 ± 0.000c	317.320 ± 0.000a
C/N	15.52 ± 0.46a	15.57 ± 1.05a	16.05 ± 0.46a	13.35 ± 0.26a
N/P	1.66 ± 0.14a	1.23 ± 0.05b	1.57 ± 0.11a	1.13 ± 0.07b
C/P	25.75 ± 1.38a	19.14 ± 0.95b	25.16 ± 1.43a	15.03 ± 0.67b
Acid phosphatase activity (U/mg·g^−1^·24h)	0.518 ± 0.007a	0.535 ± 0.017a	0.532 ± 0.018a	0.457 ± 0.015b
Alkaline phosphatase activity (U/mg·g^−1^·24h)	0.288 ± 0.009b	0.231 ± 0.008b	0.274 ± 0.008b	0.417 ± 0.007a

*SOC, soil organic carbon; TN, total nitrogen; TP, total phosphorus; TK, total potassium; AN, available nitrogen; AP, available phosphorus; AK, available potassium; C/N, the ratio of SOC and TN; C/P, the ratio of SOC and TP; N/P, the ratio of TN and TP. The different letters “a–d” indicated significant differences according to one-way ANOVA with Tukey’s test (p < 0.05)*.

Soil samples were collected in June 2017, 1week after harvesting wheat. Before planting wheat, maize and soybean residues were incorporated into the soil in the two rotation systems. A soil sampler with a diameter of 8cm and a depth of 20cm was used to collect the five soil cores as a composite sample. Three biological replicates, i.e., three composite samples, were collected to freeze on ice and immediately transported to the lab, stored at −80°C for metagenomic sequencing. The rest of the sample was stored at 4°C for further analysis.

### Determination of Soil Physical and Chemical Properties

Soil organic carbon content was determined using potassium dichromate oxidation method (Bao, 2010), soil pH was measured using a pH meter (Inesa Scientific Instrument Co., Ltd, Shanghai, China) with a soil/water ratio of 1:2.5 (w/v). Total nitrogen (TN) was measured with the Kjeldahl method (Automatic Kjeldahl Nitrogen Analyzer k1160, China). Total phosphorus (TP) was determined using alkali fusion-Mo-Sb anti-spectrophotometric method (Lu, 2000); TK was measured with alkali fusion-flame spectrophotometry (Lu, 2000). AN was extracted with potassium chloride solution and measured with spectrophotometric methods (Lu, 2000). AP was extracted using sodium hydrogen carbonate solution and measured using Mo-Sb anti-spectrophotometric method (Olsen and Sommers, 1982); AK was extracted with ammonium acetate and analyzed with flame photometry (Lu, 2000); C/N was the ratio of SOC and TN; C/P was the ratio of SOC and TP; and N/P was the ratio of TN and TP.

### Acid and Alkaline Phosphatase Activity Determination

Potential acid (ACP) and alkaline (ALP) phosphatase activity were measured following Tabatabai (1994). Briefly, 1g of fresh soil was incubated in 50ml Erlenmeyer flask with 0.2ml toluene, 4.0ml buffer (pH=6.5 for ACP, pH=11 for ALP) and 1ml 0.05 M para-nitrophenyl phosphate for 1h at 37°C. Then reactions were stopped with 1ml 0.5M CaCl_2_ and 4ml 0.5M NaOH. The absorbency of filtrate was determined at 410nm, and the enzymatic activity was expressed as μmol h^−1^ g^−1^ dry soil.

### Metagenome Sequencing

#### Soil Total DNA Isolation

Soil total DNA was isolated from a 0.5-g soil sample using PowerSoil DNA Isolation kit (MO BIO Laboratories, West Carlsbad, CA) following the procedure of the manufacturer. Extracted DNA was electrophoresed and quantified using quantitative fluorescence instrument (Qubit 2.0, Life Technologies) with the concentration ranging from 19.40ng/μl to 31.00ng/μl and stored at −20°C.

#### Library Preparation

The qualified DNA samples were sheared into many fragments of 300bp randomly using a Covaris S220 system (Covaris, Woburn, MS) and purified using Agencourt AMPure XP-nucleic acid purified beads and quantified with Qubit^®^ 2.0. DNA fragments were terminal repaired, added A tail and sequencing adaptor, purified and PCR amplified to accomplish library preparation using the NEBNext^®^ Ultra^™^ DNA Library Prep Kit for Illumina^®^. The libraries were purified once again using Agencourt AMPure XP-beads. The product within the purified library was verified by 2% agarose gel electrophoresis and quantified with the Qubit^®^ 2.0. Finally, total genomic DNA was sequenced using Illumina’s HiseqXten platform in Sangon Biotech (Shanghai) Co., Ltd.

#### Bioinformatic Processing

The original sequencing data were filtered by trimmomatic (version 0.36; Bolger et al., 2014) to remove sequences with N-base and the adapters and filter low quality bases (quality value<20). Reads less than 100bp were eliminated to get clean reads. The clean reads of each sample were assembled into the contigs with IDBA_UD (Peng et al., 2012). Prodigal (version 2.6; Liu et al., 2013) was used to predict the ORF of the splicing results, and the genes longer than 100bp were selected and translated into amino acid sequences. For the gene prediction results of each sample, CD-HIT software (version 4.6; Li and Godzik, 2006) was used to remove redundancy to obtain a non-redundant gene set. Sequences in gene sets were aligned against the Nr (NCBI non-redundant protein sequences) database using DIAMOND (version 0.8.20; Buchfink et al., 2015) with value of *E*<10^−5^, Score>60 for taxonomic annotation to get the relative abundance of species at all taxonomic levels. Sequences of protein in gene sets were aligned against the Kyoto Encyclopedia of Genes and Genomes (KEGG) database using Ghost KOALA (version 1.0; Kanehisa et al., 2016) to get the number of KO, and then connected the information of Pathway and Module to perform functional annotation. All sequences were deposited in the NCBI database with the accession numbers of SAMN11464682-11464689 and SAMN20692125-20692128.

### Statistical Analysis

The non-metric multi-dimensional scaling (NMDS) analysis was conducted with the data from MG-RAST to investigate the similarity and distances among all the soil samples using vegan package in R (version 2.0-10; Oksanen et al., 2017). Analysis of Similarity (ANOSIM) was performed using R vegan package (version 2.0-10; Oksanen et al., 2017) to measure the clustering of samples among SW and MW treatments. Statistical analysis of metabolic profile (STAMP) software (version 2.1.3) was used to evaluate statistical differences in microbial community composition in pairs (Parks et al., 2014). Linear discriminant analysis (LDA) effect size (LEfSe) was used to identify the biomarker between the rotations using LEfSe software (version 1.1.0; Segata et al., 2011). Correlation heatmap was performed with spearman analysis to build the relationship of microbial community composition and/or genes involved in P cycling with soil properties using R corrplot package (version 0.73; Wei et al., 2013).

## Results

### Soil Properties Related to Crop Rotation

Soil organic carbon contents were higher in 7years of treatments than in 2years of rotations (Table 1, *p*<0.05), indicating that 7years of rotations increased SOC. SW2 treatment decreased soil TN content compared to MW7 and SW treatments (*p*<0.05), but the rotation period did not significantly change it. Seven years of rotations decreased TP and increased TK compared with those of 2years (*p*<0.05), whereas TP and TK did not respond to different rotations. The pH values did not dramatically change under different rotations and periods. AN, AP, and AK were significantly different among rotation treatments. AP was higher in MW rotations than in SW (*p*<0.05) because of the higher P fertilizer applied in the maize season. Seven years of rotations increased C/P and N/P (*p*<0.05). SW2 treatment significantly enhanced ALP activity and decreased ACP activity compared to SW7 and MW treatments (*p*<0.05; Table 1).

### Soil Microbial Community Among Rotation Treatments

**Figure 1:**
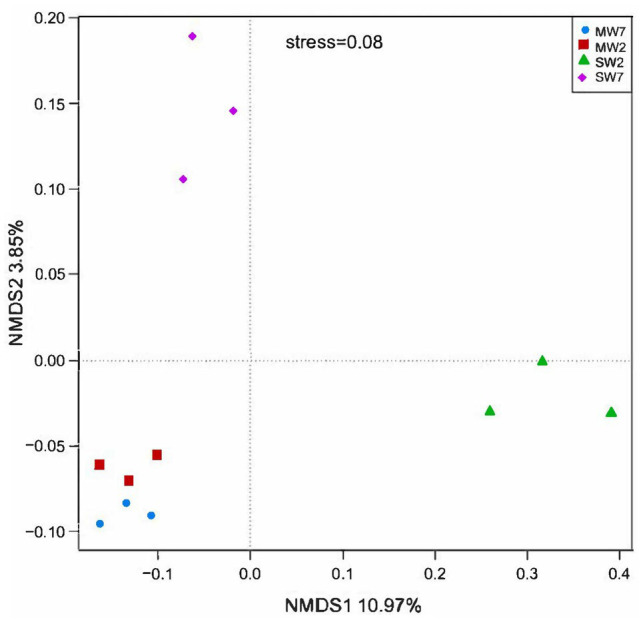
The non-metric multidimensional scaling (NMDS) analysis of bacterial phylum among different rotation treatments. MW7, maize-wheat rotations for 7years; MW2, maize-wheat rotations for 2years; SW7, soybean-wheat rotations for 7years; SW2, soybean-wheat rotations for 2years; the same as below.

Non-metric multi-dimensional scaling analysis among rotation treatments at soil microbial community level is shown in Figure 1. All the MW rotation treatments gathered more closely than they did with SW. ANOSIM with UniFrac distances revealed significant differences between the MW and SW treatments (*R*=0.667, *p*=0.03).

**Figure 2:**
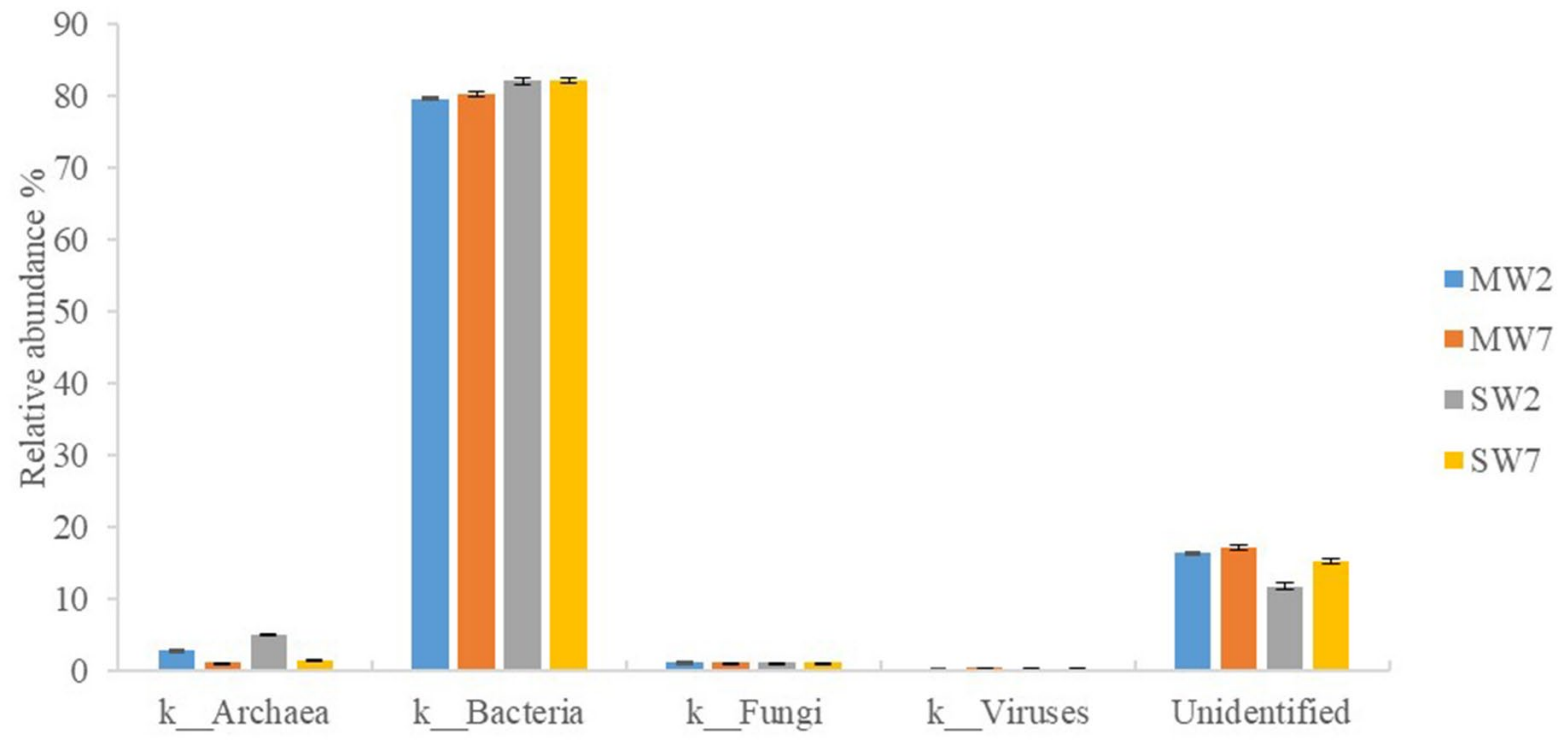
Relative abundances of soil microbial community in rotation treatments. MW2, maize-wheat rotations for 2years; MW7, maize-wheat rotations for 7years; SW2, soybean-wheat rotations for 2years; SW7, soybean-wheat rotations for 7years.

A metagenomic approach was used in our study; therefore, bacteria, fungi, archaea, and viruses were all detected in the soil microbial community. Bacteria predominated (accounting for 79.53–82.03%) in all rotations, followed by archaea (1.13–4.96%) and viruses (0.20–0.41%); eucarya accounted for only 1.09%. In addition, 12.75–18.12% of the sequences were not identified in the NR database (Figure 2).

**Figure 3:**
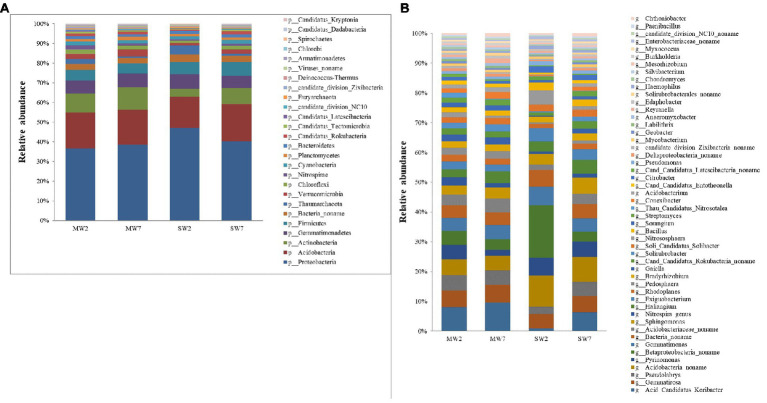
The relative abundances of soil bacterial community composition at phylum **(A)** and genus **(B)** level in the rotations.

In the present study, *Proteobacteria* was the most abundant bacteria phylum, contributing 30.57 and 37.06% in MW and SW rotations, respectively, followed by *Acidobacteria*, *Actinobacteria*, *Gemmatimonadetes*, *Firmicutes*, and *Bacteria noname*, representing 14.61, 8.55, 5.47, 4.37, and 2.27% in MW rotations and 14.56, 5.17, 5.76, 5.63, and 2.87% in SW rotations, respectively (Figure 3A). At the genus level, the bacteria communities were dominated by *Betaproteobacteria noname* (2.16 and 5.81% in MW and SW rotations, respectively), *Acidobacteria noname* (2.60 and 5.08%), *Pyrinomonas* (1.81 and 2.99%), *Gemmatimonas* (2.35 and 2.88%), *Bacteria noname* (2.19 and 2.83%), *Gemmatirosa* (3.00 and 2.75%), and *Sphingomonas* (1.73 and 2.38%; Figure 3B).

**Figure 4:**
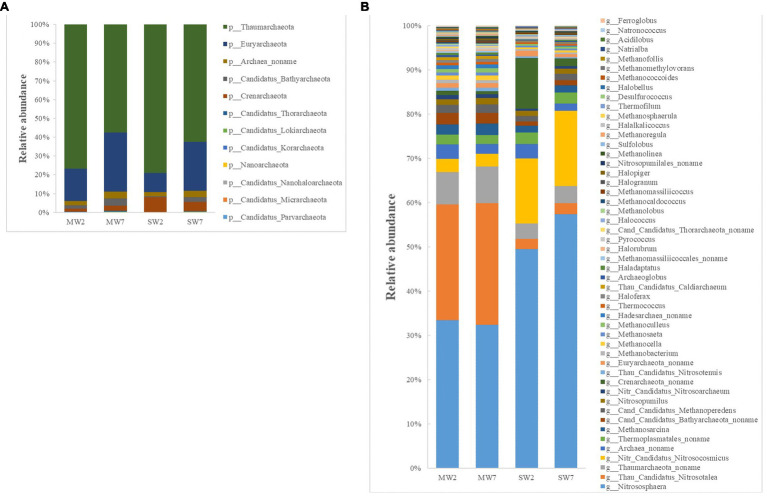
The relative abundances of soil archaea community composition at phylum **(A)** and genus **(B)** level in the rotations.

The first four phyla of archaea were *Thaumarchaeota* (accounting for 57–79% of all archaea), *Euryarchaeota* (10–32%), *Crenarchaeota* (2.0–8.0%), and *Archaea noname* (2.0–4.0%; Figure 4A). The genera of archaea differed between SW and MW rotations. The relative abundances of *Nitrososphaera*, *Nitr Candidatus Nitrosocosmicus* and *Crenarchaeota noname* in SW were much higher than those in MW rotations, whereas *Thau Candidatus Nitrosotalea*, *Thaumarchaeota noname*, and *Cand Candidatus Bathyarchaeota_ noname* were dramatically less than in MW rotation (Figure 4B).

**Figure 5:**
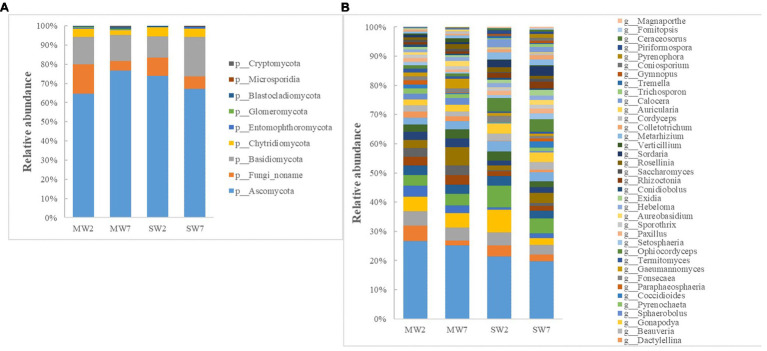
The relative abundances of soil fungi community composition at phylum **(A)** and genus **(B)** level in the rotations.

The major phyla of fungi were *Ascomycota*, *Basidiomycota*, *Fungi noname*, and *Chytridiomycota*, representing 65–77%, 11–21%, 5–15%, and 2–5% of the relative abundance in all treatments (Figure 5A). At the genus level, *Ustilaginoidea*, *Fusarium*, *Pseudogymnoascus*, *Drechmeria*, *Aspergillus*, and *Mucor* predominated in soil fungi. The abundances of *Ustilaginoidea*, *Penicillium*, *Aspergillus*, and *Talaromyces* in SW rotations were significantly less than in MW treatments, whereas *Mucor* was the opposite (Figure 5B).

**Figure 6:**
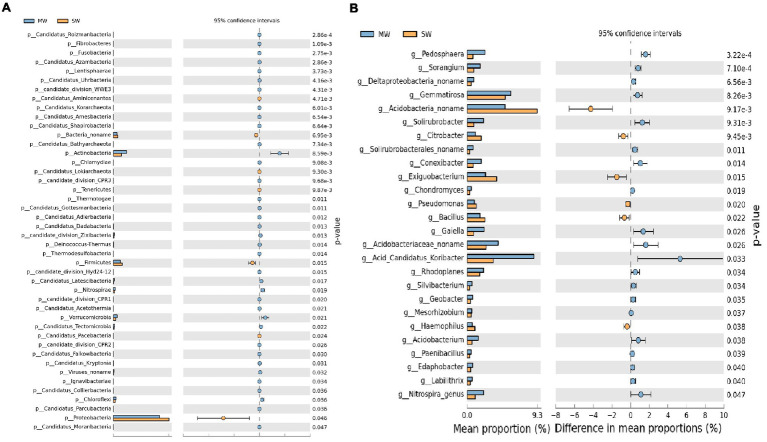
Stamp analysis of **(A)** phylum and **(B)** genus of soil bacterial community between SW and MW rotations. SW, soybean-wheat rotations; MW, maize-wheat rotations.

Stamp analysis showed that among the top 15 phyla with relative abundances more than 1%, *Actinobacteria*, *Verrucomicrobia*, and *Chloroflexi* enriched in MW rotations, while *Proteobacteria*, *Firmicutes*, and *Bacteroidetes* enriched in SW rotations (Figure 6A, *p*<0.05). Eleven genera with relative abundances more than 1% showed dramatic differences between SW and MW rotations. *Acidobacteria noname*, *Exiguobacterium*, and *Bacillus* enriched in SW rotations. *Acid Candidatus Koribacter*, *Gemmatirosa*, *Acidobacteriaceae noname*, *Pedosphaera*, *Gaiella*, and *Solirubrobacter* were enriched in MW rotations (Figure 6B, *p*<0.05). LEfSe was used to evaluate which microbiome attributes differ significantly among rotations; however, no biomarkers were identified (Supplementary Figure S1).

**Figure 7:**
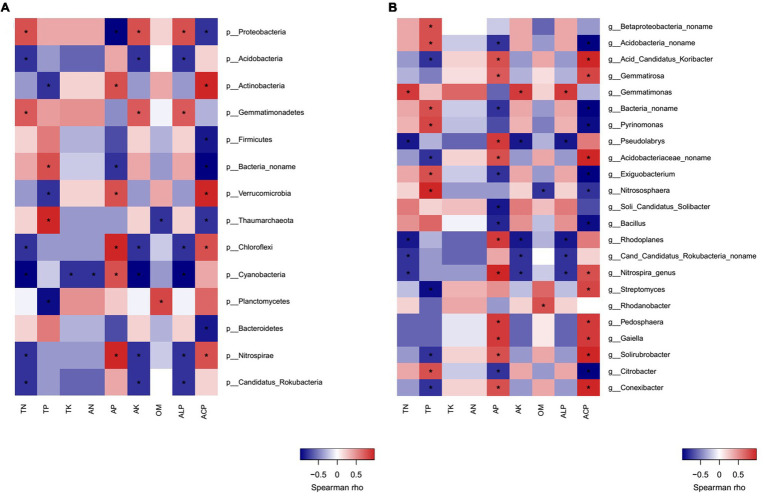
Correlation heatmap analysis of phylum **(A)** and genus **(B)** of bacterial community composition with soil properties in MW and SW rotations.

Correlation heatmap analysis showed that there were significant positive or negative correlations between several bacterial phyla and genera and soil properties (Figures 7A,B). Strong positive correlations were observed between *Proteobacteria/Gemmatimonadetes* and TN, AK, ALP; *Actinobacteria* and AP, ACP; *Verrucomicrobia/Chloroflexi/Nitrospirae* and AP, ACP. On the other hand, the negative correlations between *Proteobacteria/Bacteria no name* and AP, ACP; *Acidobacteria/Nitrospirae/Candidatus Rokubacteria/Chloroflexi* and TN, AK, and ALP; *Actinobacteria/Verrucomicrobia/Planctomycetes* and TP; *Firmicutes/Bacteroidetes* and ACP; *Cyanobacteria* and TN, AN, TK, AK, and ALP were observed (Figure 7A).

### Effects of Rotations on Genes Involved in P Transformation

The genes mainly consisted of such functions as mineralization of organic P, solubilization of inorganic P, P regulation, P uptake and transport system, etc. In total, 2.88% of sequences referred to genes coding for enzymes involved in soil microbial P cycling.

**Figure 8:**
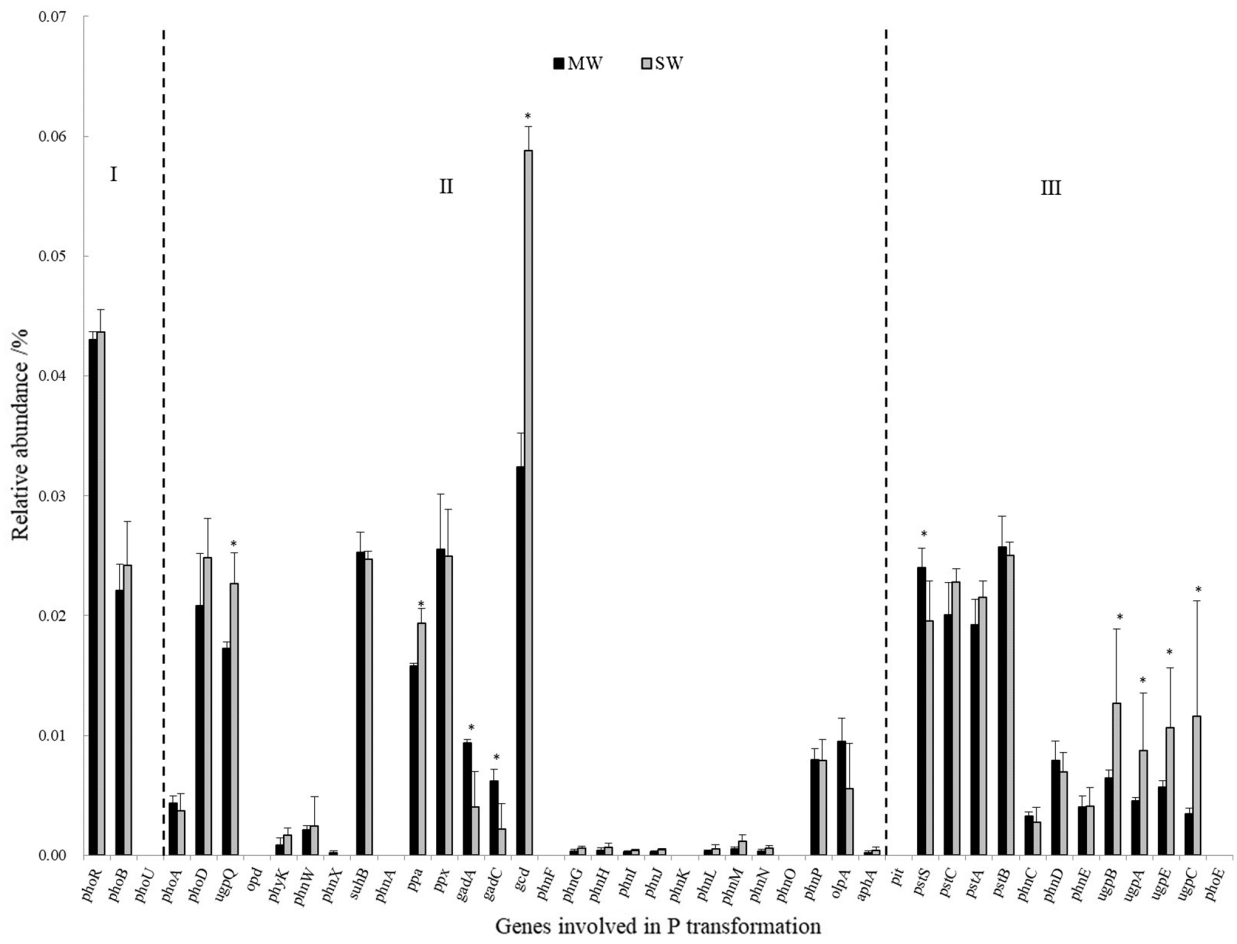
The relative abundances of genes involved in P transformation between SW and MW rotations. SW, soybean-wheat rotations; MW, maize-wheat rotations; I, Genes coding for P-starvation response regulation; II, Genes coding for inorganic P-solubilization and organic P-mineralization; III, Genes coding for P-uptake and transport. Asterisk represents the significant differences in the relative abundance of genes involved in P transformation at *p*<0.05 between MW and SW rotations.

**Figure 9:**
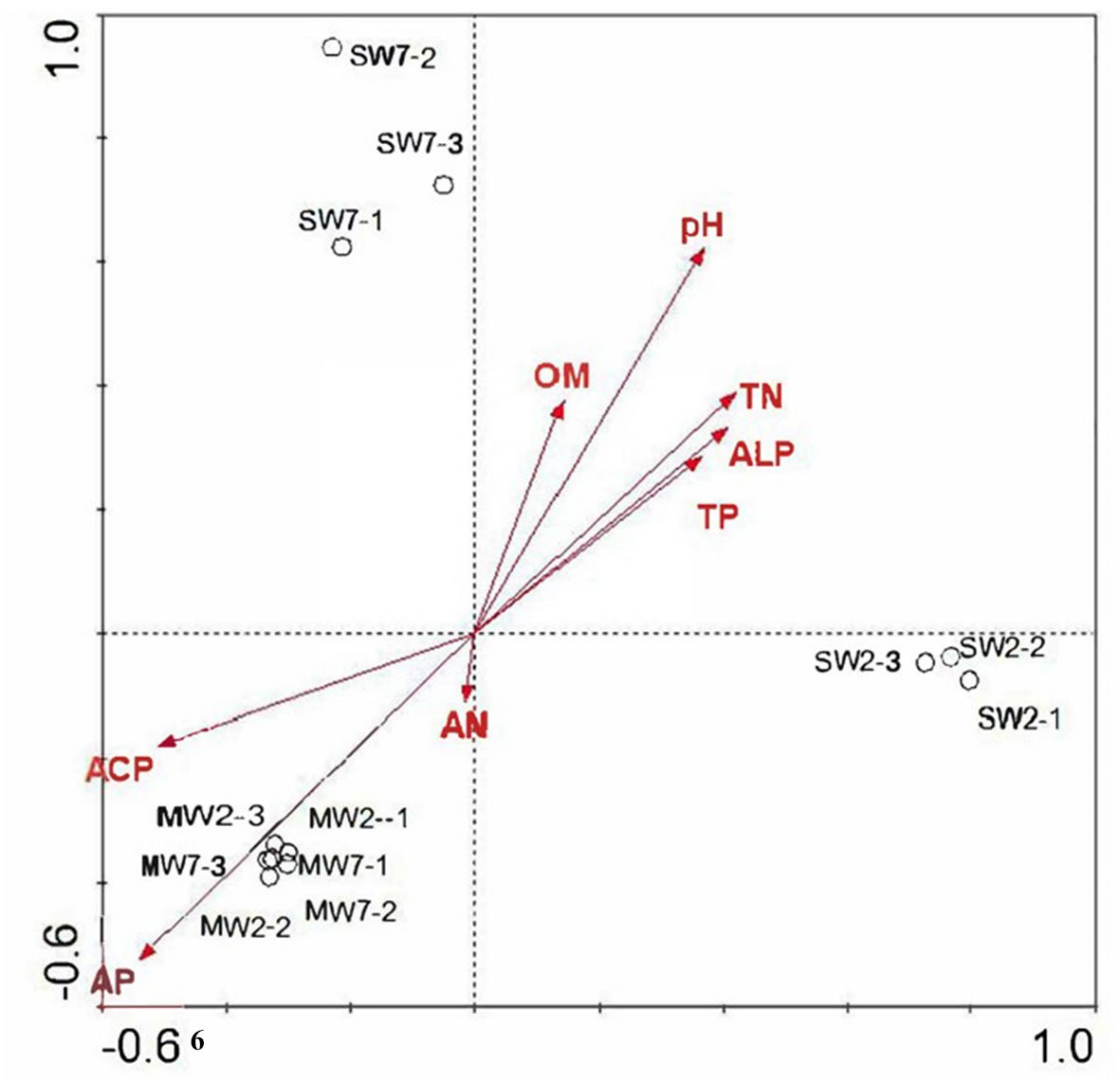
Redundancy analysis of soil microbial functional genes involved in P transformation among treatments according to soil properties.

Genes coding for glucose dehydrogenase (GDH, *gcd*) dominated in all treatments, followed by genes of phosphate regulon (*phoB*, *phoR*), organic P mineralization (*phoD*, *ugpQ*, *suhB*, *ppa*, *ppx*), and phosphate transport system (*pstABCS*). Whereas the low relative abundances of genes referring to sn-glycerol 3-phosphate transport system (*ugpABCE*), phosphate transport system (*phnCDE*), and part of phosphatase (*phyK*, *olpA*, *phoA*, and *phoB*), especially C-P lyase core reaction (*phnGHIJLM*), were detected in all rotations. The gene of *gcd* was 84.38% more in SW rotations than in MW (*p*<0.05), which contributed 0.059 and 0.032% of all genes in these two rotations, respectively. On the contrary, *gadA* and *gadC* significantly displayed less abundances in SW rotations than in MW (Figure 8), revealing the dramatic differences in inorganic P transformation between MW and SW rotations. For organic P transformation genes, the abundance of *ugpQ* was significantly higher in SW rotations than in MW. Significant differences were observed for *pstS* and *ugpABCE* genes. There were only 3 genes (*phnN* and *pstBC*) showing significant differences between 2 and 7years of treatments (Supplementary Figure S2). We further performed redundancy analysis (RDA) to determine the contributions of environmental variables to functional genes involved in P cycling (Figure 9). The results indicated that AP and ACP contributed to the P cycling in MW rotations, while the other soil properties except for AN contributed more in SW rotations.

### Effects of Rotation on the Microbial Community Harboring Functional Genes of P Cycling

**Figure 10:**
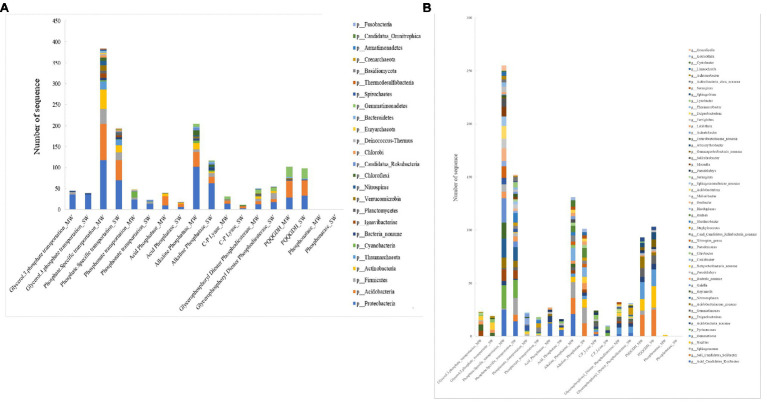
The taxonomic analysis of soil bacterial community involved in P cycling functional genes and at **(A)** phylum and **(B)** genus among treatments.

The microbial community involved in P transformation was assigned at the phylum and genus levels (Figure 10). All functional genes involved in P turnover were divided into several major groups: glycerol-3-phosphate transportation, phosphate specific transportation, phosphonate transportation, acid phosphatase, alkaline phosphatase, C-P lyase, glycerophosphoryl diester phosphodiesterase, phosphonatase, and GDH. Phylum of *Proteobacteria* harbored all groups of investigated genes, especially genes coding for phosphate-specific transportation, alkaline phosphatase, and GDH. The relative abundances of *Proteobacteria* harboring functions of phosphate-specific transportation and alkaline phosphatase in MW rotations were 68.6 and 61.9% more than in SW rotations (*p*<0.05), whereas *Proteobacteria* harboring functions of GDH was 12.1% less than in SW rotations. *Acidobacteria* covered most of the investigated genes except for those coding for glycerol-3-phosphate transportation, phosphonate transportation, and phosphonatase. The relative abundance of *Acidobacteria* harboring functions of phosphate-specific transportation and alkaline phosphatase and GDH in MW rotations was 79.2% (*p*<0.05), 140% (*p*<0.05), and 5.4% more than in SW rotations, respectively. Such genera as *Acid Candidatus Koribacter*, *Soli Candidatus Solibacter*, *Sphingomonas*, *Gemmatirosa*, *Pyrinomonas*, *Acidobacteria noname*, *Exiguobacterium*, and *Gemmatimonas* played important roles in phosphate-specific transportation and alkaline phosphatase and GDH (Figure 10B).

The NMDS analysis of soil microbial functional genes involved in P cycling among rotation treatments is shown in Supplementary Figure S3. Similarly, all the MW rotations gathered more closely with one another than they did with SW rotations. The ANOSIM indicated that crop rotations also significantly shifted the soil microbial function involved in P transformation (*R*=1.0, *p*=0.01). SW2 treatments were separated from those of SW7.

## Discussion

### Effects of Rotations on Soil Microbial Community Structure

Non-metric multi-dimensional scaling and ANOSIM analysis revealed that rotation significantly altered the soil bacterial community. Our result is in accordance with the findings of Zhang et al. (2017) and Guo et al. (2020). Whereas rotation for 7years did not affect soil microbial community compared to those for 2years.

Crop rotations influenced soil bacterial community composition, which corroborates with the result of Ai et al. (2018). The rotation system may influence the soil microbial community by soil properties, crop types, or plant residue (Li et al., 2019). The higher P applied *via* fertilizer in the maize growth period compared with that of soybean resulted in higher AP content in MW rotation. Fertilizer application rate is highly dependent on crop type, thus MW rotation increased AP content (Table 1), the relative abundance of *Bacillus* genus was negatively related to AP in the present study (Figure 7B), which was in accordance with the previous study (Huang et al., 2020). The lower AP content stimulated *Bacillus* to solubilize non-available P into orthophosphate to provide AP for crops (Tye et al., 2002). This is partly explained by the enrichment of *Bacillus* in SW rotations. The enrichment of *Bacillus* in SW rotation was also related to crop type. For example, compared to maize preceding soybean significantly increased the population density of *Bacillus* in oilseed rape rhizosphere (Yang et al., 2020). *Bacillus* is preferable to enrich the soybean rhizosphere soil compared to maize, due to its N-fixing and P-solubilizing capacity (Aloo et al., 2019). *Bacillus* was one of the degraders of bean plant residue (Ortiz-Cornejo et al., 2017), soybean residue presumably induced its enrichment in SW rotations. The recent studies indicated that soil pH played a key role in altering the microbial community (Long et al., 2018; Wang et al., 2018); however, crop rotation did not change pH values a lot in the present study. Therefore, the regulation effects of pH were not observed. Due to the N-fixing function of soybean, SW rotation increased TN content, the abundances of *Nitrospira* and *Rhodoplanes* genera were negatively related to the TN. This resulted in lower abundances in SW rotations compared to those of MW.

In the present study, *Proteobacteria*, *Acidobacteria*, *Actinobacteria*, *Gemmatimonadetes*, and *Firmicutes* were the predominant bacterial phyla, although these groups are commonly found in wheat soils (Souza et al., 2013; Wen et al., 2016; Chen et al., 2017). The positive correlation between *Proteobacteria* and TN, *Actinobacteria*, *Verrucomicrobia*, *Chloroflexi*, and AP resulted in the significant enrichments of them in SW and MW treatments, respectively. MW rotation increased *Actinobacteria*, *Verrucomicrobia*, and *Chloroflexi* compared to SW rotation in the present study. Similar to our study, Souza et al. (2013) also reported that *Verrucomicrobia* was enhanced in MW rotations. Different from our results, crop rotation [soybean/maize (summer)/wheat/lupine/oat (winter)] reduced the relative abundance of *Actinomycetales* (*Actinobacteria*) compared with crop succession [soybean (summer)/wheat (winter); Souza et al., 2013]. *Actinobacteria* had been shown to be enriched in the maize rhizosphere (Peiffer et al., 2013). *Actinobacteria* and *Chloroflexi* play important roles in decomposition of organic materials such as cellulose derived from corn stover (Merklein et al., 2014; Pepe-Ranney et al., 2016), their higher abundances in MW treatments may be related to the maize residue in the soils. In addition, isoflavonoids, a key root exudate component in soybean, probably inhibited the growth of *Actinobacteria* (White et al., 2017), which may also explain the lower abundance of *Actinobacteria* in SW rotations. *Acidobacteria* and *Chloroflexi* grow in soils with low available nutrient so that they are classified as slow-growing oligotrophs (Fierer et al., 2007). MW rotations increased the relative abundance of *Chloroflexi* and AP content and reduced TN content compared with SW rotations, which indicated that it is not P but N that drives the presence of *Chloroflexi* phylum. Generally, only a slight effect was imposed on the soil microbial community composition in our study, one possible explanation is that the number of plant species in rotation (only 2 crops in each rotation) in our experiment was not high enough (D’Acunto et al., 2018).

The relative abundance of soil fungi in our study is less than in the previous study (Jacquiod et al., 2016). On one hand, soil fungal abundance was associated with a large amount of fertilizer input (Pan et al., 2020), decreased soil acidity, and C/N (Ning et al., 2020). A large amount of inorganic fertilization and high C/N in our study (13.35–16.05) probably inhibited the growth of soil fungi (Ji et al., 2020). On the other hand, the composition and relative abundance of the microbial community in metagenomic sequencing depend on the soil DNA extraction method, reference database, and annotation procedure (Jacquiod et al., 2016). We extracted the soil DNA using the PowerSoil DNA Isolation kit (MO BIO laboratories, West Carlsbad, CA), which is a direct method. Generally, the direct extraction method had no negative effect on the relative proportion of total eukaryote and microeukaryote. We used the reference database of nr (non-redundant protein sequences), which is officially collected by NCBI. It includes all translation sequences of non-redundant GenBank CDs, protein data bank (PDB) protein database, Swissprot protein database, and protein sequences from protein information resource (PIR) and Protein Research Foundation (PRF) databases. The database gave a somewhat lower number of classes of soil microbial community and had underrepresentation of fungal sequences compared to M5NR (Jacquiod et al., 2016).

### Effects of Crop Rotations on Genes Involved in P Cycling

Little is known on the effect of rotation on functional genes involved in P transformation. The genes involved in microbial P transformation consisted of three groups, including genes involved in P-starvation response regulation, inorganic P-solubilization and organic P-mineralization, and P-uptake and transport. Dissolving inorganic P and mineralizing organic P to provide AP for the crops is one of the important contributions of the soil microbial community for sustainable agricultural development, so genes encoding for inorganic P-solubilization and organic P-mineralization were discussed here.

#### Effects of Crop Rotation on Genes Involved in Inorganic P Solubilization

In the present study, the predominant genes coding for inorganic P solubilization were inorganic pyrophosphatase (*ppa*), exopolyphosphatase (*ppx*), *gcd*, *gadA*, and *gadC*. The much higher relative content of *gcd* gene indicated that mineral-P was rich in both rotation soils and higher potential for P solubilization and revealed that the strategy to acquire P for wheat is to dissolve inorganic P (Bergkemper et al., 2016; Liang et al., 2020). SW rotations dramatically increased the relative abundances of *gcd* gene than MW, one explanation for this observation may be the significant negative relationship between *gcd* gene and AP content (Supplementary Figure S4). The previous study reported that Olsen-P was an important P chemistry predictor of the P cycling functional gene composition (Liu et al., 2018). Similarly, *gcd* gene abundance was negatively correlated with soil CaCl_2_-P, citrate-P, enzyme-P, and HCl-P (Gao and DeLuca, 2018). Another explanation is due to more *Bacillus* in the SW rotation (Figure 6B), which had P solubilizing ability in soil.

It has been reported that glucose could be oxidated into gluconic acid by GDH, which requires pyrroloquinoline quinone (PQQ) as a cofactor (Ben Farhat et al., 2013). Both genes together encode the enzyme of PQQGDH. The gene of *gcd* codes for GDH. The pqq operon consists of at least 5 *pqq* genes designated as *pqqABCDE*. Among these, PqqC catalyzes the final step of biosynthesis of PQQGDH, and was designated as a molecular marker for phosphate-solubilizing capacity (Meyer et al., 2011). The reduced P input with organic–inorganic fertilization slightly increased *pqqC* gene abundance (Bi et al., 2020). PqqE is a functional radical SAM enzyme capable of catalyzing reductive cleavage of SAM to methionine and 5′-deoxyadenosine and plays an important role in phosphate solubilizing (Ludueña et al., 2017a). Ludueña et al. (2017b) reported that lower P in soil promoted *pqqE* gene expression. In our study, the relative abundance of genes related to the PQQ cofactor was lower than those coding for GDH. This suggests a partly PQQ-independent activation of GDH (Grafe et al., 2018). It is worth noting that *gadA* and *gadC* genes showed the opposite pattern compared with the *gcd* gene; that is, significantly higher abundance in MW rotation than in SW rotation (Figure 8). The former two genes encode for gluconate 2-dehydrogenase. The opposite pattern suggested that SW rotation had higher potential of producing gluconate to dissolve soil bound inorganic P.

#### Effects of Crop Rotations on Genes Involved in Organic P Mineralization

Genes involved in organic P mineralization consist of C-P lyase (*phnW* and *phnP*), phosphomonoesterase (*suhB*), phosphodiesterase (*ugpQ*), and phosphotriesterase (*opd*). Among these, phosphomonoesterase also includes alkaline phosphatase (*phoA* and *phoD*) and acid phosphatase (*olpA* and *aphA*; Zhou et al., 2018). Three genes of *suhB*, *phoD*, and *ugpQ* dominated in all treatments (Figure 8 and Supplementary Figure S2), while no genes encoding phosphotriesterase (*opd* and *php*) and C-P lyase (*phnK*) were observed. This suggested that the high potential for organic P mineralization did not derive from phosphonate but from phosphomonoester and phosphodiester, revealing the main composition of organic P in the soils. The phosphonates were generally reported to be components of fungal glycoproteins, exopolysaccharides, and membrane phosphonolipids (McGrath et al., 2013). The much smaller abundance of the compound demonstrated much lower relative abundance of fungi, the speculation nicely confirmed previous data of the much lower ratio of fungi in the whole microbial composition (Figure 2).

Significant difference not for *suhB* (coding for phosphomonoester), but for *ugpQ* (coding for phosphodiester) between SW and MW treatments was observed. Our result showed the stronger potential of phosphodiester mineralization in SW rotations, which was in accordance with the previous study (Zhou et al., 2018). According to the binding of substrate, the planting of legumes possibly increased soil phosphodiester content and showed higher potential for phosphodiester mineralization compared with maize crop (Zhou et al., 2018). However, to date, the distribution of *suhB* and *ugpQ* in soil environment is poorly documented.

Genes coding for alkaline phosphatase were more than those for acid phosphatase. This may be due to the neutral pH values in soils (Grafe et al., 2018). Among genes coding for alkaline phosphatase, *phoX* was not detected, and the abundance of *phoD* was higher than *phoA* among all rotations, consistent with the previous studies, so *phoD* was the most common typical alkaline phosphatase gene harbored by soil bacteria (Tan et al., 2013). No significant differences in the relative abundances of *phoD* between rotation were observed, irrespective of lasting time of rotation in our study (Figure 8 and Supplementary Figure S2). Similarly, *phoD* gene also showed no significant differences in land uses of grassland soil, bare fallow soil, and arable soil (Neal et al., 2017). The previous study reported that *phoD* gene abundance varied in the soils with different crops (Liu et al., 2021). The gene *phoD* was also influenced mainly by fertilization patterns. Generally, *phoD* gene abundance may be inhibited by high P application, but enhanced by P deficiency (Chen et al., 2017). The same wheat crop and fertilization in our field suggested the non-significant differences between the two rotations. Although rotation altered soil AP, genes encoding phosphatase did not differ between rotations. Similarly, significantly different AP did not alter the *phoD* gene abundance in a rice field (Long et al., 2018). One explanation is that *phoD*-harboring bacteria showed high diversity and respond differently to both rotations (Ragot et al., 2015; Fraser et al., 2017; Liu et al., 2021). Furthermore, global patterns of phosphatase activity in natural soils demonstrated that TN was also the significant factor driving the distribution of phosphatase activity (Margalef et al., 2017). Thus, *phoD* was driven by the interaction of TN and AP. In addition, the *phoD* gene encoded alkaline phosphatase (Fraser et al., 2015a,b; Acuña et al., 2016), crop rotations did not alter alkaline phosphatase activity, and consequently, did not alter *phoD* gene abundance.

In general, significantly much higher abundances of *gcd* and *ugpQ* in SW rotation compared to MW rotation indicated higher capacity of organic P mineralization and inorganic P solubilization in SW treatment. Given the similar TP and significantly lower AP in SW rotation, the results revealed that wheat and microorganisms in SW rotation had stronger abilities of P uptake. Consequently, SW rotation improved the P solubilization and uptake of microorganism and wheat, and produced an ecological advantage in agriculture.

### Taxonomic Assignments of Key Functional Genes Involved in P Cycling

Taxonomic assignment of genes involved in P transformation emphasized the predominance of *Proteobacteria* in rotations. *Acidobacteria* and *Gemmatimonadetes* also played key roles in P turnover especially in phosphate uptake systems, C-P lyase, organic phosphorus mineralization, and inorganic phosphorus solubilization.

SW rotation decreased the relative abundance of *Proteobacteria, Acidobacteria, Firmicutes*, and *Actinobacteria* harboring phosphate transportation genes and phosphatase (Figure 10A), while increased the *Proteobacteria* harboring glycerophosphoryl diester phosphodiesterase and PQQGDH. This may be due to soil microorganisms with the capacity of solubilization of inorganic bound P mainly composed of *Proteobacteria*, *Acidobacteria*, and *Gemmatimonadetes* phyla. Genes involved in P transformation were mainly harbored by genera of *Acid Candidatus Koribacter*, *Soli Candidatus Solibacter*, *Sphingomonas*, *Gemmatirosa*, and *Pyrinomonas*. *Bacillus* and *Gemmatimonadetes* also own the *gcd* gene (Figure 10B). SW treatments decreased the genera harboring phosphate specific transportation, phosphatase genes, and increased the genera harboring PQQGDH gene. Because many studies found that *Bacillus*, *Pseudomonas*, *Azotobacter*, *Burkholderia*, *Rhizobium*, *Clostridium*, *Serratia*, *Acinetobacter*, *Enterobacter*, and *Klebsiella* could dissolve inorganic P in soil (Barroso and Nahas, 2005; Browne et al., 2009; Uribe et al., 2010; Singh and Prakash, 2012; Liu et al., 2015), *Enterobacter* sp. Fs-11 belonging to rhizobacterium had the ability of phosphate solubilizing and plant growth promoting and owned the gene of *pqqE* (Shahid et al., 2012). *Rhizobiales* (in *Proteobacteria*) contributed a lot in inorganic phosphorus solubilization in the P-rich soil (Bergkemper et al., 2016) and paddy soil (Long et al., 2018). *Arthrobacter*, *Bacillus*, *Brevibacterium*, and *Streptomyces* were the predominant genera of inorganic phosphate solubilizing bacteria in long-term different fertilization regimes (Zheng et al., 2017).

## Conclusion

This field experiment demonstrated that crop species in rotations had different influences on soil microbial community and function referring to P cycling. SW rotation increased the relative abundances of *Proteobacteria*, *Firmicutes*, and *Bacteroidetes* phylum, and decreased *Actinobacteria*, *Verrucomicrobia*, and *Chloroflexi*. The relative abundances of *Proteobacteria* harboring functions of phosphate-specific transportation and alkaline phosphatase in MW rotations were more than in SW rotations (*p*<0.05), whereas *Proteobacteria* harboring functions of GDH were less than in SW rotations. SW rotation increased the relative abundance of *Bacillus* genus, increased the genes of *gcd* and *ugpQ*, and reduced *gadA* and *gadC*. Our results helped to explain the mechanism that SW rotation showed the higher potential of P uptake of wheat and soil microbial community. These results emphasize the need to select proper crop species in agroecosystems and to reduce the fertilization in agroecological strategies in order to sustainably maintain the function of agroecosystems. Better understanding of the interactions of crop management practices, soil, and the composition and function of microbial communities is of significance for selecting appropriate crops to maintain the sustainable development of agriculture.

## Data Availability Statement

The datasets presented in this study can be found in online repositories. The names of the repository/repositories and accession number(s) can be found at:

https://www.ncbi.nlm.nih.gov/, SAMN11464682-11464689.

https://www.ncbi.nlm.nih.gov/, SAMN20692125-20692128.

## Author Contributions

FW and LK conceived the study. HY and YaX contributed to the data analysis of bioinformatics. MS and LH contributed to the field experiment and soil sampling. HY contributed to draft the article. YuX, FW, and LK contributed to critically review and edit the manuscript. All authors contributed to the article and approved the submitted version.

## Funding

This work was supported by the National Natural Science Foundation of China (41807039) and the China Postdoctoral Science Foundation (2016M602168).

## Conflict of Interest

The authors declare that the research was conducted in the absence of any commercial or financial relationships that could be construed as a potential conflict of interest.

## Publisher’s Note

All claims expressed in this article are solely those of the authors and do not necessarily represent those of their affiliated organizations, or those of the publisher, the editors and the reviewers. Any product that may be evaluated in this article, or claim that may be made by its manufacturer, is not guaranteed or endorsed by the publisher.
