# Supplementary material for: Effects of Rotations With Legume on Soil Functional Microbial Communities Involved in Phosphorus Transformation
Source: Front Microbiol. 2021 Sep 30;12:661100. doi: 10.3389/fmicb.2021.661100 (PMC8519609; doi:10.3389/fmicb.2021.661100)

**Supporting Informations**

**Effects of rotations with legume on** **soil** **functional microbial communities involved in phosphorus transformation**

Hui Yu^1^, Fenghua Wang^1,2,*^, Yangyang Xie^1^, Minmin Shao^3^, Ling Huang^3^, Yuxin Xu^1^, Lingrang Kong^2,*^

^1^National Engineering Laboratory for Efficient Utilization of Soil and Fertilizer Resources, College of Resources and the Environment, Shandong Agricultural University, 61 Daizong Road, Taian 271018, China; ^2^State Key Laboratory of Crop Biology, College of Agronomy, Shandong Agricultural University, 61 Daizong Road, Taian 271018, China. ^3^Jining Academy of Agricultural Sciences, 9 Jidai Road, Jining, 272009, China.

*Corresponding authors: Lingrang Kong, Fenghua Wang

E-mail: [lkong@sdau.edu.cn](mailto:lkong@sdau.edu.cn) (L. Kong), [wfh@sdau.edu.cn](mailto:wfh@sdau.edu.cn) (F. Wang),

**Figure S1.** Linear discriminant analysis (LDA) effect size (LEfSE) circular diagram of bacterial taxa that differentiate among rotation treatments.

**Figure S2.** The relative abundances of genes involved in P transformation between 2 and 7 years of rotations. Ⅰ, Genes coding for P-starvation response regulation; Ⅱ, Genes coding for inorganic P-solubilization and organic P-mineralization; Ⅲ, Genes coding for P-uptake and transport. Asterisk represents the significant differences in the relative abundance of genes involved in P transformation at p < 0.05 between 2 and 7 years of rotations.

**Figure S3.** The non-metric multi-dimensional scaling (NMDS) analysis of soil P cycling functional genes among different rotation treatments.

**Figure S4.** The correlative heatmap between P cycling functional genes and soil properties.

**Fig. S1**


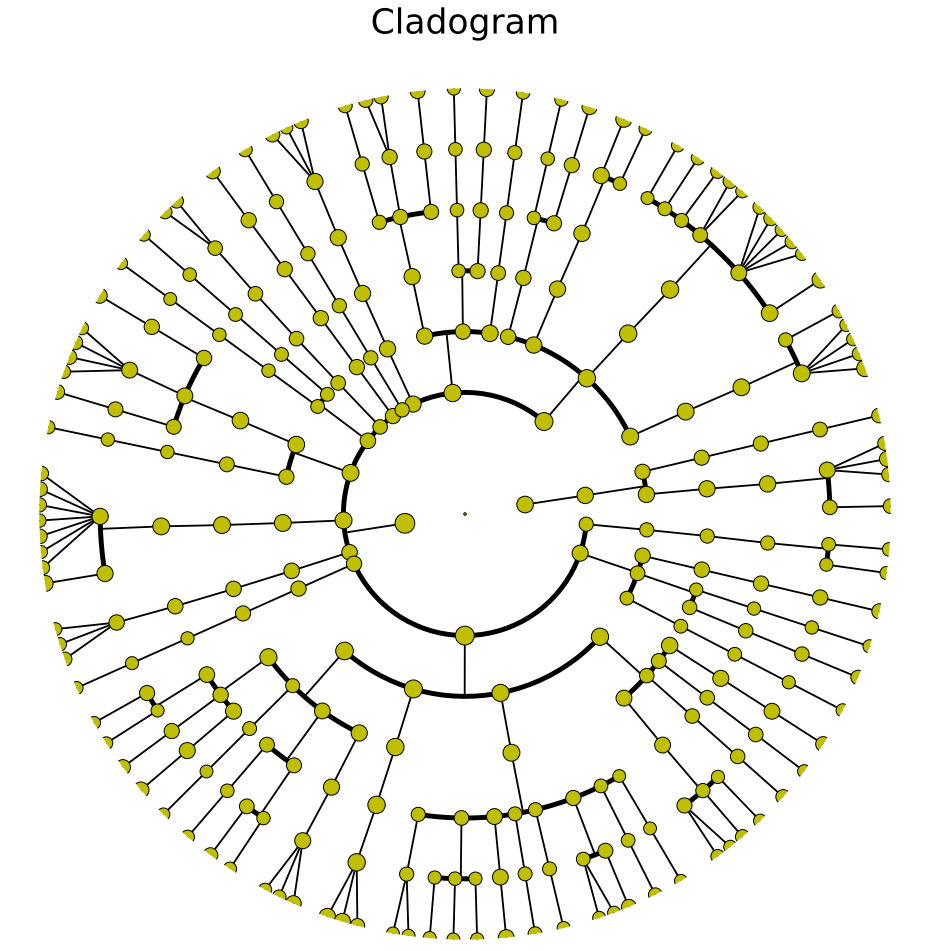


**Fig. S2**

**
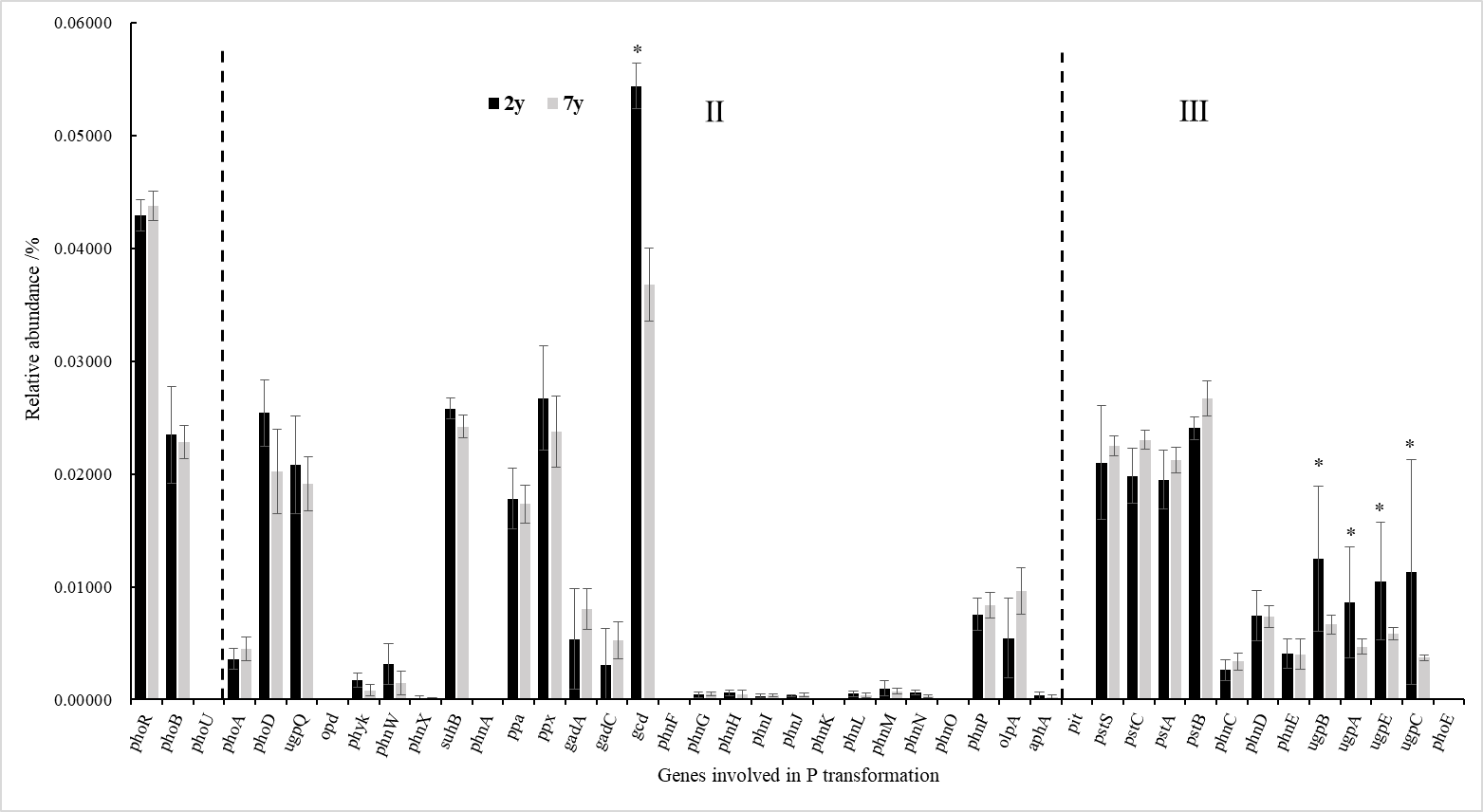
**

**Fig. S3**


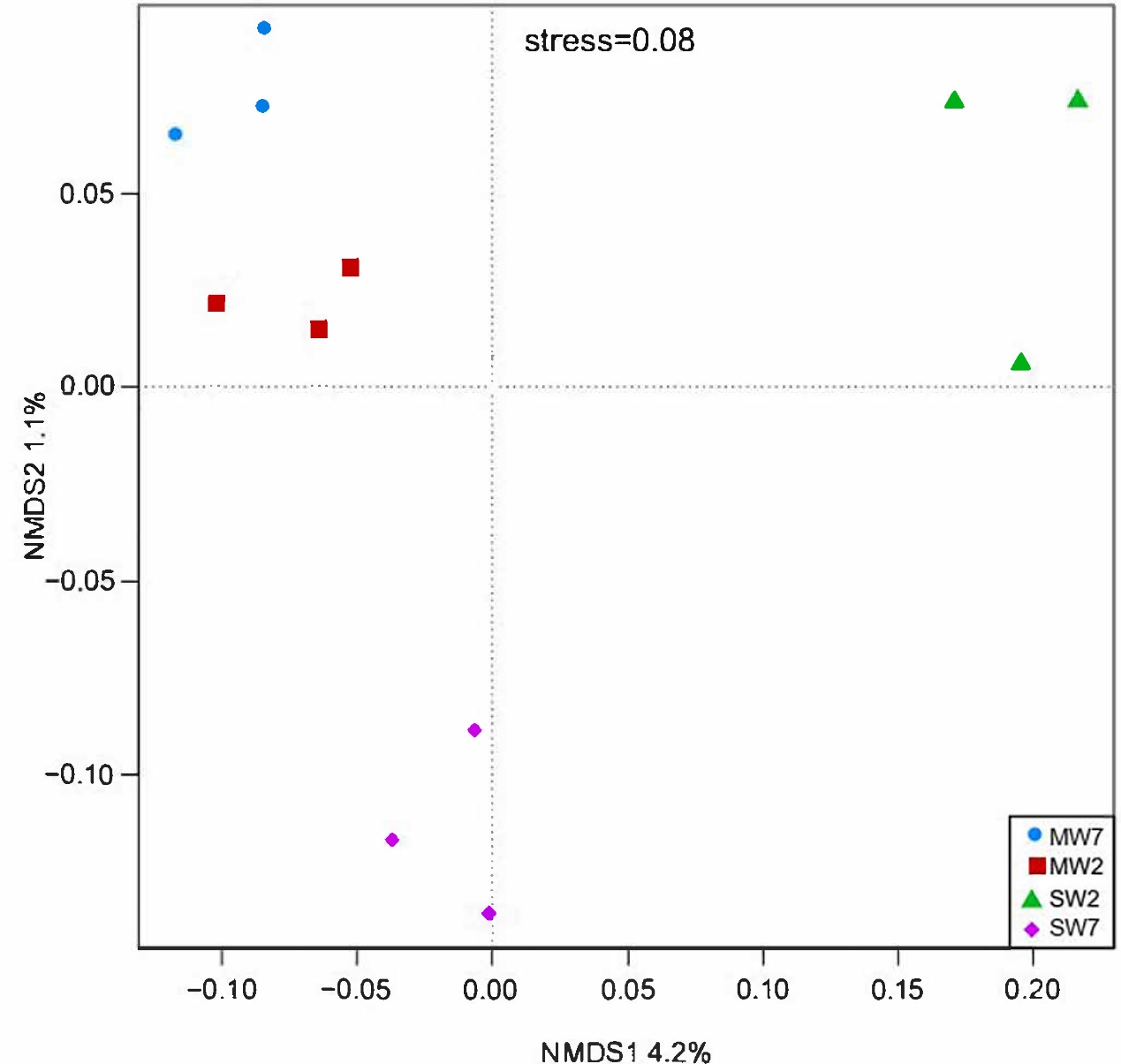


**Fig. S4**


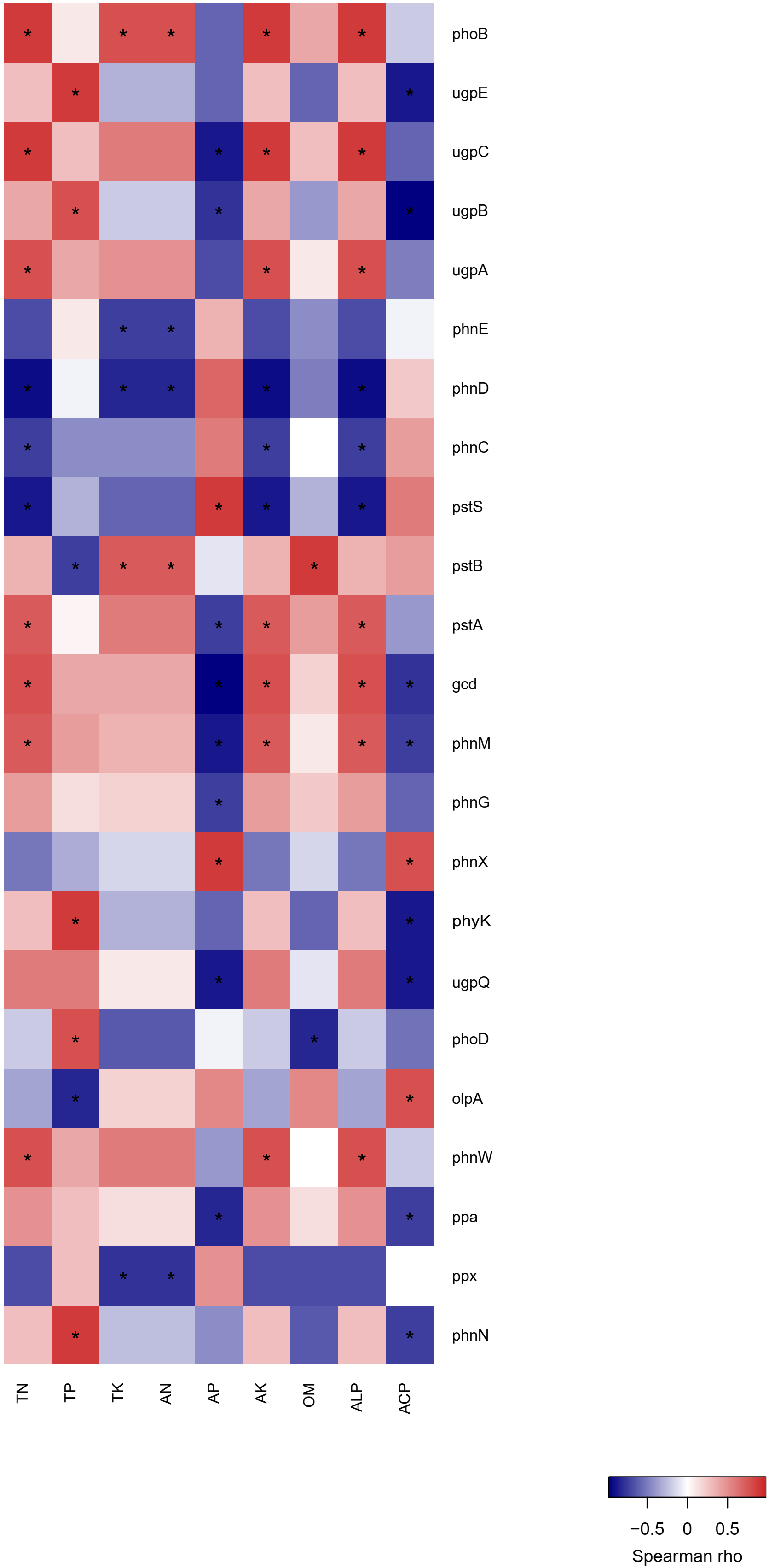

Supplement: Supplementary file 1 [file Data_Sheet_1.DOCX]
